# Developing a hybrid time-series artificial intelligence model to forecast energy use in buildings

**DOI:** 10.1038/s41598-022-19935-6

**Published:** 2022-09-21

**Authors:** Ngoc-Tri Ngo, Anh-Duc Pham, Thi Thu Ha Truong, Ngoc-Son Truong, Nhat-To Huynh

**Affiliations:** 1 Faculty of Project Management, The University of Danang - University of Science and Technology, 54 Nguyen Luong Bang, Da Nang, Vietnam; 2 Department of Civil Engineering, The University of Danang-University of Technology and Education, 48 Cao Thang Street, Da Nang City, Vietnam

**Keywords:** Civil engineering, Electrical and electronic engineering

## Abstract

The development of a reliable energy use prediction model is still difficult due to the inherent complex pattern of energy use data. There are few studies developing a prediction model for the one-day-ahead energy use prediction in buildings and optimizing the hyperparameters of a prediction model is necessary. This study aimed to propose a hybrid artificial intelligence model for forecasting one-day ahead time-series energy consumption in buildings. The proposed model was developed based on the integration of the Seasonal Autoregressive integrated Moving average, the Firefly-inspired Optimization algorithm, and the support vector Regression (SAMFOR). A large dataset of energy consumption in 30-min intervals, temporal data, and weather data from six real-world buildings in Vietnam was used to train and test the model. Sensitivity analyses were performed to identify appropriate model inputs. Comparison results show that the SAMFOR model was more effective than the others such as the seasonal autoregressive integrated moving average (SARIMA) and support vector regression (SVR), SARIMA-SVR, and random forests (RF) models. Evaluation results on real-world building depicted that the proposed SAMFOR model achieved the highest accuracy with the root-mean-square error (RMSE) of 1.77 kWh in, mean absolute percentage error (MAPE) of 9.56%, and correlation coefficient (R) of 0.914. The comparison results confirmed that the SAMFOR model was effective for forecasting one-day-ahead energy consumption. The study contributes to (1) the knowledge domain by proposing the hybrid SAMFOR model for forecasting energy consumption in buildings; and (2) the state of practice by providing building managers or users with a powerful tool for analyzing and improving building energy performance.

## Introduction

The building sector is one of the significant energy consumers, which consumes about 40 percent of the world's energy use and 30 percent of carbon dioxide generation^1–3^. Energy demand is still increasing due to rapid economic development and urban expansion^4,5^. Sustainable development of energy is an important concern for many countries^6^. Occupant behaviors in buildings may force energy efficiency and can save about 50 percent of the total global energy usage^7^. Energy use reduction in buildings is beneficial to society in terms of economy and ecology.

Future prediction of energy data is a method of projecting future data based on historical time-series data. Energy consumption prediction of households is difficult because it is affected uncertainly by occupant`s behaviors^8^. Because the nature of the energy use exhibits the complex and seasonal pattern, the unreliable forecast may result in an additional production or waste of resources^9^. For example, the prediction of the household electricity use is vital for smart grid development and the energy market^10^. Therefore, a reliable prediction method is important for proper investment planning of energy generation and distribution^6^. Accurate prediction results are valuable for decision-makers in planning energy demand and in saving energy proactively.

Building energy data is recognized as time-series data that vary along with various timestamps such as daily, hourly, 30-min, 15-min, or 5-min intervals. Statistics-based methods and machine learning (ML) methods have been developed for predicting time-series data. An autoregressive integrated moving average (ARIMA) is an example of powerful statistical method^11^. In the ARIMA model, an autoregressive (AR) part regresses on previous values, a moving average (MA) part regresses on a purely random process while an integrated part renders the data via differencing^12^. For time-series data with seasonality herein, the ARIMA is not effective to capture data patterns. Meanwhile, a seasonal autoregressive integrated moving average (SARIMA) is used for energy demand prediction^13^ and sales forecasting^14^. However, these two models are suitable for modeling the linear relationship between the predictors and dependent variables.

Deb et al.^15^ and Wang and Srinivasan^16^ have reviewed the artificial intelligence (AI) techniques for building energy prediction. The gradual maturity of AI can create an opportunity in recording big data and understanding the insights behind data. AI-based modes have been developed recently to improve the performance of regression problems^17^ such as the prediction of the hydro-power production capacity^18^. They have confirmed the powerful approach for solving complex problems^19–21^ such as the prediction of stock market indices^22^, and hydro-power production capacity^18^. Seyedzadeh et al.^23^ developed a machine learning model for predicting building energy loads to support building design and retrofit planning.

Artificial neural networks (ANNs) and support vector regression (SVR) models are two widely used models in the energy domain. The ANNs was integrated with the particle swarm optimization (PSO) in predicting building electricity consumption^24^. The integration of ANNs and ARIMA models was proposed for predicting time-series data^25^. Although the ANNs model can obtain relatively high predictive accuracy, it has several limitations such as the difficulty of controlling variables, overfitting issues, and uncertain solutions^12^.

The SVR models have been used in solving regression problems. For example, the SVR was used to forecast the hourly cooling energy demand in office buildings^26^ and to predict the water temperature of the reservoir^27^. The SVR was combined with the genetic algorithm (GA) to forecast energy use^28^. However, the SVR is relatively slow in dealing with huge data^29^ and has a high computational burden^30^. The least-squares SVR (LSSVR)^31^, an improved variant of the SVR, is also widely used for prediction problems because it can reduce the computational effort^32^. The LSSVR model is more effective than the SVR model because the LSSVR model solves linear equations rather than quadratic programming problems and employs a squared loss function^31^.

However, optimizing hyperparameters of the LSSVR model is concerned by researchers to enhance the predictive performance in predicting energy consumption. Fine-tuning LSSVR`s hyperparameters is an optimization problem that can be solved by the nature-inspired metaheuristic optimization algorithms^33^, differential evolution (DE) algorithm^34^, PSO^35^, and firefly algorithm (FA)^36^. Because of the capabilities of automatic subdivision and addressing of multimodality, the FA has been confirmed as an effective optimization algorithm^33^. It is widely used to solve various problems in many domains^36,37^. Therefore, the FA was used to optimize the LSSVR`s hyperparameters in this study.

To the best of the authors’ knowledge, there are few studies performing the one-day-ahead energy use prediction in buildings in the literature. The one-day-ahead energy consumption with 30-min intervals was used because it can provide insights for users to adjust actions on reducing their energy use. Studies on selecting optimal inputs for prediction models are still limited in literature. In addition, the length of the learning data has an impact on the performance of prediction models. Optimizing hyperparameters of prediction models is necessary. These are the research gaps in previous studies. All these concerns are addressed in this study. This work aims to propose a hybrid artificial intelligence prediction model for forecasting one-day ahead time-series energy consumption in buildings toward sustainable development. The proposed model was developed based on the integration of the Seasonal Autoregressive integrated Moving average, the Firefly-inspired Optimization algorithm, and the support vector Regression (SAMFOR).

In the proposed SAMFOR model, hyperparameters of the SAMFOR model will be optimized by the FA to enhance the predictive accuracy. As part of this study, selecting appropriate inputs and size of the learning data will be performed in section “Sensitivity analysis” (sensitivity analysis) and they can improve the predictive accuracy of the prediction models. A large dataset of energy consumption was collected in a 30-min interval for two years from six buildings in Danang city in Vietnam. This dataset was used to train and test the performance of the proposed model. By validating with various datasets, the proposed model shows generalization in doing an energy use prediction in buildings.

The first contribution of this work is the proposed effective prediction model in accurately forecasting the one-day-ahead energy consumption with 30-min intervals in buildings. Compared to the traditional method and individual machine learning models, the proposed hybrid SAMFOR model enables us to learn the linear and nonlinear profiles of building energy use, which can significantly improve the prediction accuracy. In addition, the model can consider the impact of the temporal data (i.e., day of the week and hour of the day), weather data (i.e., outdoor temperature and humidity), and historical energy data as the inputs for the future energy use prediction in buildings. For practical contribution, the prediction results provide building owners, building managers, and users with insights and references to adjust their behavior and reduce energy use and energy management.

The remainder of this paper is organized as follows. Section “Literature review” presents the literature review, and section “Hybrid prediction model based on SARIMA and optimized SVR” describes the proposed model and its implementation. Section “Data collection and analytical results” presents the dataset and analytical results. Section “Conclusions” provides concluding remarks and future work.

## Literature review

Various prediction models were developed based on a single machine learning model^38^, ensemble ML models such as XGboost, the feedforward deep networks (FDN)^10^, AdaBoost^39^, ensemble models^40^, and hybrid ML models^9^. The SVR model was applied to implement energy use prediction and diagnosis of public buildings^41^. Energy-saving solutions in buildings have attracted the interest and concerns of various researchers^10,42^. Engie North America has applied AI and machine learning to enhance data governance and quality^43^. They proposed automated and an analytics system to assess energy use data. Their system can improve risk determination and provide flexible pricing strategies. Besides, Patel^44^ has an in-depth analysis of artificial intelligence’s role in the power sector. He mentioned unbiased and technically sound assessment of AI methods is extremely important to the industry.

Day-ahead subentry energy use in the building sector has been predicted using fuzzy C-means clustering and nonlinear regression in^39^. Particularly, hourly heating ventilation air-conditioning (HVAC) subentry and hourly socket subentry in an office building were used to validate their method. Pham et al.^42^ presented an application of the random forests (RF)—based ML model for forecasting short-term electricity use patterns in buildings. Five sets of time series data of energy consumption were applied to build and test the RF model in comparison against the M5 model trees and random tree. Various scenarios were used to test the energy prediction accuracy of the RF model and it confirmed that the RF`s outstanding performance with the enhancement up to 49.95 percent in the mean absolute error compared to the base models in the 12-steps-ahead electricity use^42^.

Chen et al.^10^ proposed an ensemble ML that combines the FDN and extreme gradient boosting (XGboost) forest for predicting annual building electricity use. The XGBoost was developed by Chen et al.^11^ that combines a set of regression trees^45^. The number of boosts and maximum tree depth is two main hyper-parameters in the XGboost model that represent the number of regression trees and the maximum tree depth of each single regression tree developed in the XGboost model, respectively. The structures of these above-mentioned base models were designed optimally and determined by varying combinations of their parameters. The ensemble model can improve the predictive accuracy with 30% in the root mean square error.

Ngo^46^ has investigated the effectiveness of various single and ensemble approaches for building energy simulation and prediction. Individual ML models consist of ANNs, SVR, CART, and LR while the ensemble models were developed upon these individual ML models in the voting, bagging, and stacking methods. The ensemble models yielded 0.98–0.99 in the correlation coefficient and 6.17–12.93 percent in the mean absolute percentage error (MAPE). The ensemble ANNs with the bagging method obtained the best performance among all investigated models.

Kaytez^6^ hybridized ARIMA and least-squares support vector machine (LSSVM) to produce a prediction model of long-term energy use. The ARIMA`s parameters were adjusted to predict the trend component in time-series energy data while the SVM was to model the residual component. Historical data of gross electricity generation, population, installed capacity, import, export, and total subscribership were collected from 1970 to 2017 and used as predictors for long-term energy consumption prediction. The multiple linear regression model and a single ARIMA model were used as a baseline for performance comparison. Their findings indicated that the ARIMA-LSSVM was more realistic and reliable than the baseline model such as the multiple linear regression and single ARIMA^6^.

Li et al.^9^ integrated the sine cosine algorithm (SCA) and SVM to propose the SCA-SVM model for short-term electricity demand prediction in which the SCA was used to optimize the penalty factor and the kernel function of the SVM. For pre-processing time-series data, Fourier decomposition was utilized to extract the fluctuation characteristics, and data seasonality was eliminated before feeding to the prediction model. The evaluation results from four datasets revealed that the proposed hybrid model is powerful in short-term electricity prediction. Liu et al.^39^ used holt-winters and extreme learning machines to predict residential electricity consumption.

Shen et al.^8^ improved the performance SVR model in forecasting electricity consumption in a residential building under various intervention strategies. The Gaussian radial basis function (RBF) was applied in the kernel function and fine-tuned by a GA. Historical data of occupant behaviours, personality traits, demographic and building attributes, and weather conditions were used as inputs for the SVR model for future energy use prediction. The model can determine the suitable intervention option and forecast the maximum energy savings in households. The results revealed that an average saving amount was 12.1 percent in electricity consumption with the traditional behavioural intervention^8^.

Some hybrid prediction approaches have been introduced recently for long-term and short-term energy consumption such as the hybridization of an autoregressive integrated moving average ARIMA and LSSVM by Kaytez^6^, the integration of the sine cosine algorithm (SCA), and support vector machine (SVM) by Li et al.^9^, GA-based improved SVR^8^. The effectiveness of these methods has been discussed. However, the appropriate inputs for prediction models and the length of the learning data used for training prediction models have few been considered in the literature.

Although various AI techniques have been proposed to develop the prediction models in previous works, few studies have combined linear ML models and nonlinear ML models. Based on the reviewed AI techniques and their power, this study proposed an AI-based hybrid prediction model to forecast the next 24-h energy use profile in buildings. The proposed model combined the SARIMA model, the SVR model, and the FA—based optimization algorithm. The proposed SAMFOR model is expected to address the above-mentioned issues. The large dataset from various buildings in Danang city in Vietnam will be used as case studies to evaluate the effectiveness of the proposed model. The details of the model will be presented in section “Hybrid prediction model based on SARIMA and optimized SVR”.

## Hybrid prediction model based on SARIMA and optimized SVR

Although machine learning techniques have been applied widely for modeling building energy performance, the prediction of the building energy consumption is a challenging task because its profile varies quite randomly. To respond to the complex patterns in building energy consumption, this study proposed the hybrid model that combines the linear time-series prediction model and the nonlinear time-series prediction model. The hybrid model enables effectively modeling the linear and nonlinear energy consumption.

### Linear time-series prediction model

SRIMA is the most commonly used linear model for predicting seasonal time series data in both academic research and industrial applications^14^. In this study, the SARIMA model was used to capture the linear patterns in the energy use profile. Seasonal AR and MA terms in the SARIMA model predict energy consumption in building *y*_*t*_ by using data values and errors at previous periods with lags that are multiples of the seasonality length *S*. The SARIMA(*p, d, q*) × (*P, D, Q*)_*S*_, is a multiplicative model that consists of nonseasonal and seasonal elements. Equation (1) presents the mathematical expression of the SARIMA model as described in^13,47^. The terms of the model are expressed in^11^.

1 $$\theta_{p} (B)\Theta_{P} \left( {B^{S} } \right)(1 - B)^{d} \left( {1 - B^{S} } \right)^{D} y_{t} = w_{q} (B)W_{Q} \left( {B^{S} } \right)\alpha_{t}$$

where *p* is the nonseasonal AR; *d* is nonseasonal differencing; *q* is the nonseasonal MA; *P* is the seasonal AR; *D* is seasonal differencing; *Q* is the seasonal MA order; *S* is the season length; *B* is the backward shift operator; *w*_*q*_*(B)*, *θ*_*p*_*(B)*, *Θ*_*P*_(*B*^*S*^)*,* and *W*_*Q*_(*B*^*S*^) are polynomials in *B*; *y*_*t*_ is the actual value at the time *t*; *α*_*t*_ is the estimated residual at the time *t*; *d, q, P, D, Q* are integers.

The SARIMA model forecasts the next 24-h energy consumption in buildings with the assumption of a linear relationship among historical data. To enhance the predictive performance, the integration of the SVR and FA has been combined with the SARIMA model to develop the hybrid model for energy consumption prediction. The SVR and FA theories were presented in section “Nonlinear time-series prediction model”.

### Nonlinear time-series prediction model

#### Support vector machine for regression

**Figure 1:**
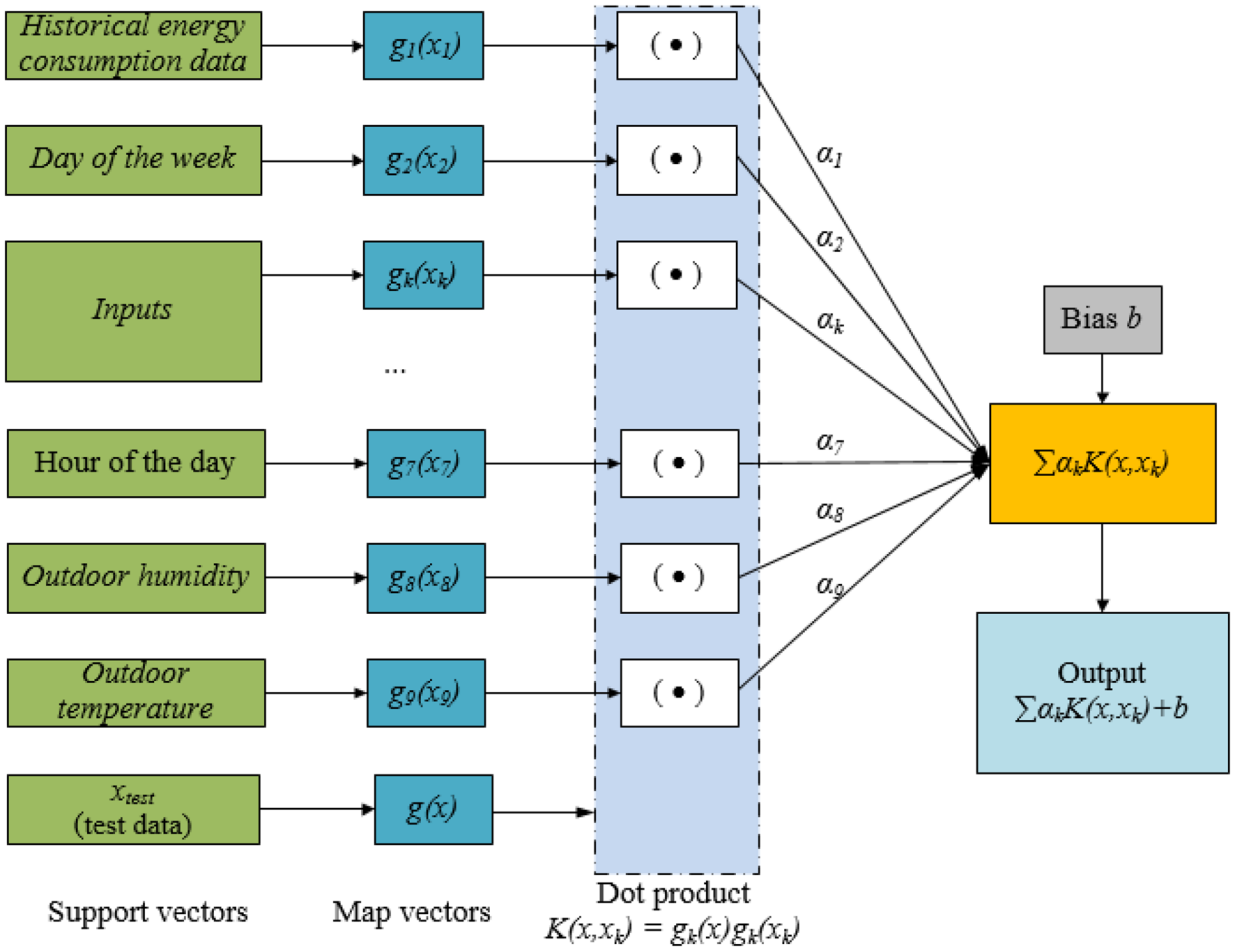
Structure of SVR model for regression.

The support vector regression (SVR)^48^ is a supervised ML technique based on the statistical learning theory and the principle of structural risk minimization. Figure 1 presents the structure of the SVR model for regression. For enhancing efficiency and generalization capacity, the LSSVR was developed^31^ to deal with large data sets such as building energy consumption data. Given a training data set $\left\{ {x_{k} ,y_{k} } \right\}_{k = 1}^{N}$, function estimation using LSSVR is formulated as an optimization problem, as expressed in Eq. (2).

2 $$\mathop {\min }\limits_{\omega ,b,e} J(\omega ,e) = \frac{1}{2}\left\| \omega \right\|^{2} + \frac{1}{2}C\sum\limits_{k = 1}^{N} {e_{k}^{2} } ;\;{\text{subject}}\;{\text{to}}\;y_{k} = \left\langle {\omega ,\varphi (x_{k} )} \right\rangle + b + e_{k} ,\;\; \, k = 1, \ldots N$$

where *J(ω,e)* denotes the optimization function, *ω* denotes the linear approximator parameter, $e_{k} \in R$ denote error variables, $C \ge 0$ denotes a regularization constant specifying the constant representing the trade-off between empirical error and function flatness, *x*_*k*_ denotes input patterns, *y*_*k*_ denotes prediction outputs, and *N* denotes the sample size.

The Gaussian radial basis function (RBF) was used as a kernel function. The FA was used to fine-tune the hyperparameters of the SVR model including *C* and the RBF width *σ*, which helps to improve the predictive accuracy of the proposed model.

#### Firefly algorithm

The FA^49^, is a nature-inspired metaheuristic algorithm that is inspired by the flashing behavior of fireflies. The FA is effective to identify the global optima and local optima. The FA operation is based on three main principles: a firefly is attracted to other fireflies; the brightness of fireflies impacts its attractiveness regarding the distance among fireflies, and the brightness is affected by the search space of the optimization problems. An optimal solution is affected by the movement of fireflies during the optimization process. The movement of a firefly is expressed as Eq. (3).

3 $$x_{i}^{t + 1} = x_{i}^{t} + \beta_{0} e^{{ - \gamma r_{ij}^{2} }} \left( {x_{j}^{t} - x_{i}^{t} } \right) + \alpha^{t} \theta_{i}^{t}$$

where $x_{i}^{t + 1}$ is the position of the *i*th firefly; $x_{i}^{t}$ is the position of the *i*th firefly; $x_{j}^{t}$ is the position of the *j*th firefly; *α*^*t*^ is a randomization parameter; and $\theta_{i}^{t}$ is random numbers; *β*_*0*_ is the attractiveness at *r* = 0; *r* is the distance between the firefly and other fireflies.

**Figure 2:**
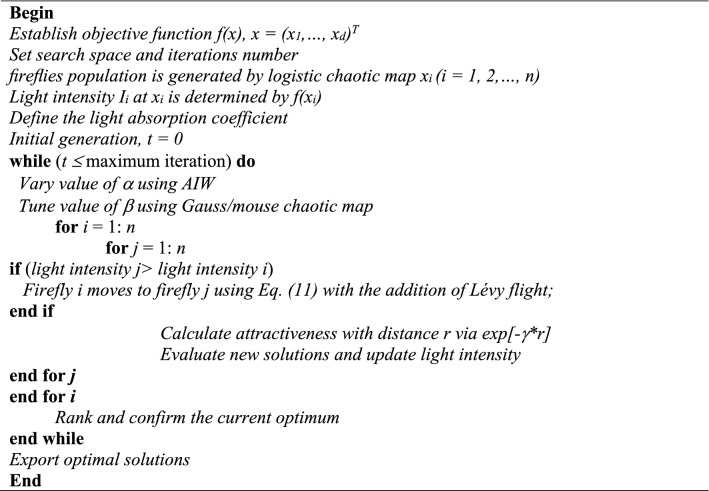
Pseudocode of modified firefly algorithm.

Details of the FA were presented in^49^. To improve the performance of the FA, this study adopted the modified version of FA that was developed by Chou and Ngo^50^. Figure 2 reveals the pseudocode of the modified FA. A Gauss/mouse map was applied to change an attractiveness parameter while a logistic map in the modified FA generates a diverse population of fireflies. The adaptive inertia weight (AIW) was adopted to vary the randomization parameter α, which can improve the local exploitation and the global exploration during the progress of the optimization process. Moreover, Lévy flights facilitate local exploitation. Figure 2 reveals the pseudocode of the modified FA.

### Learning and test process of the proposed SAMFOR model

**Figure 3:**
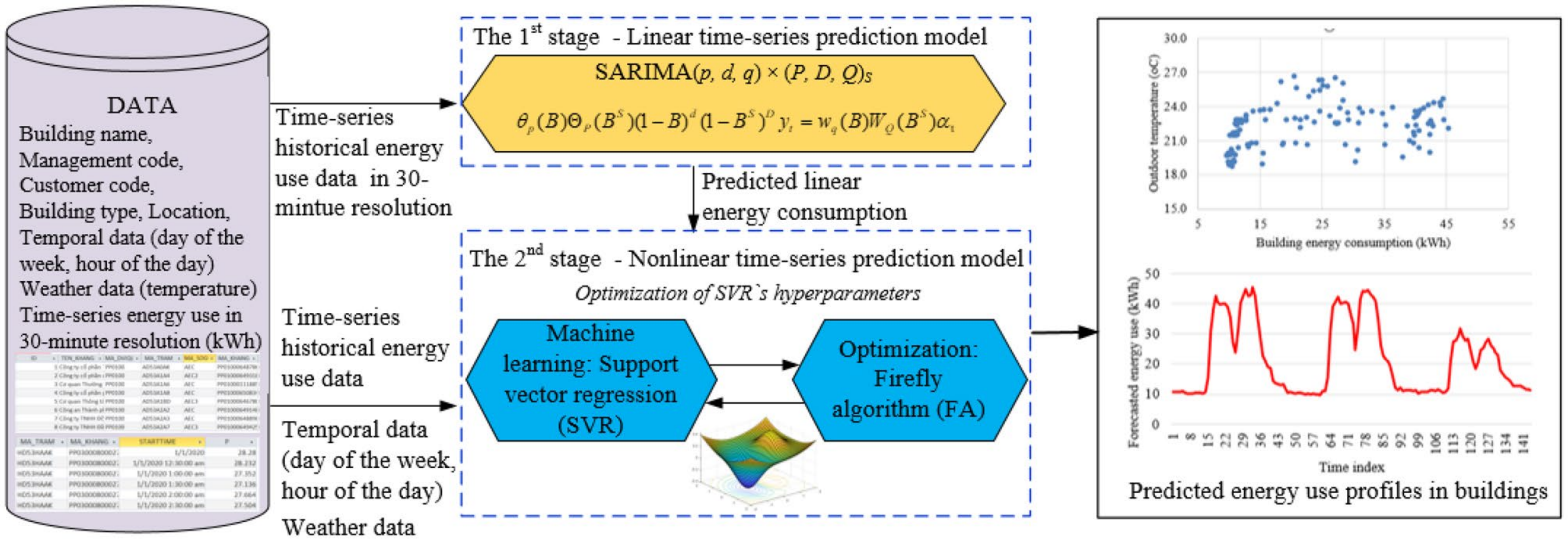
Flowchart of the proposed SAMFOR model.

Figure 3 depicts the two-stage flowchart of the proposed SAMFOR model in predicting time-series energy consumption in buildings. Data description is be presented in section “Energy consumption data and weather data”. The building energy consumption data were constituted by linear and nonlinear parts, as illustrated in Eq. (4). In the 1st stage, the collected historical energy use was fed into the linear time-series prediction model (i.e., SARIMA) to predict the linear component of the building energy consumption data. For the 2nd stage, the nonlinear time-series prediction model (i.e., FA-SVR) was used to predict the nonlinear component of the building energy consumption.

4 $$Y_{t} = L_{t} + N_{t}$$

where *Y*_*t*_ represents the building energy consumption data, *L*_*t*_ and *N*_*t*_ represent the linear part and the nonlinear part in building energy consumption data, respectively.

Equation (5) depicts the predictive results obtained by the SARIMA model in which the linear part in building energy consumption data is modeled as the predicted building energy consumption $\left( {\hat{L}_{t} } \right)$ and residual values (*R*_*t*_). As shown in Fig. 3, the inputs in the 1st stage are only time-series historical building energy consumption data which was in the 30-min resolution.

5 $$L_{t} = \hat{L}_{t} + R_{t}$$

where $\hat{L}_{t}$ are the forecasted values by the SARIMA model and *R*_*t*_ are the residual values.

The final prediction results of future building energy consumption were performed in the 2nd stage by the FA-SVR model. Inputs for this stage consists of the forecasted values $\hat{L}_{t}$, time-series historical building energy consumption, temporal data (i.e., day of the week—*DoW* and hour of the day—*HoD*), and weather data (i.e., outdoor temperature and humidity data). Therefore, the forecasted results of building energy consumption were presented as Eq. (6).

6 $$Y_{t} = \left( {DoW_{t} ,HoD_{t} ,T_{t} ,H_{t} ,\hat{L}_{t} ,Y_{t - 1} ,Y_{t - 2} , \ldots ,Y_{t - lag} } \right)$$

where *DoW*_*t*_ is the day of the week; *HoD*_*t*_ is the hour of the day; *T*_*t*_ is outdoor temperature; *H*_*t*_ is outdoor humidity data; *Y*_*t−1*_* is* building energy consumption value at the time *t−1; Y*_*t−lag*_ is the time (*t−lag).*

**Figure 4:**
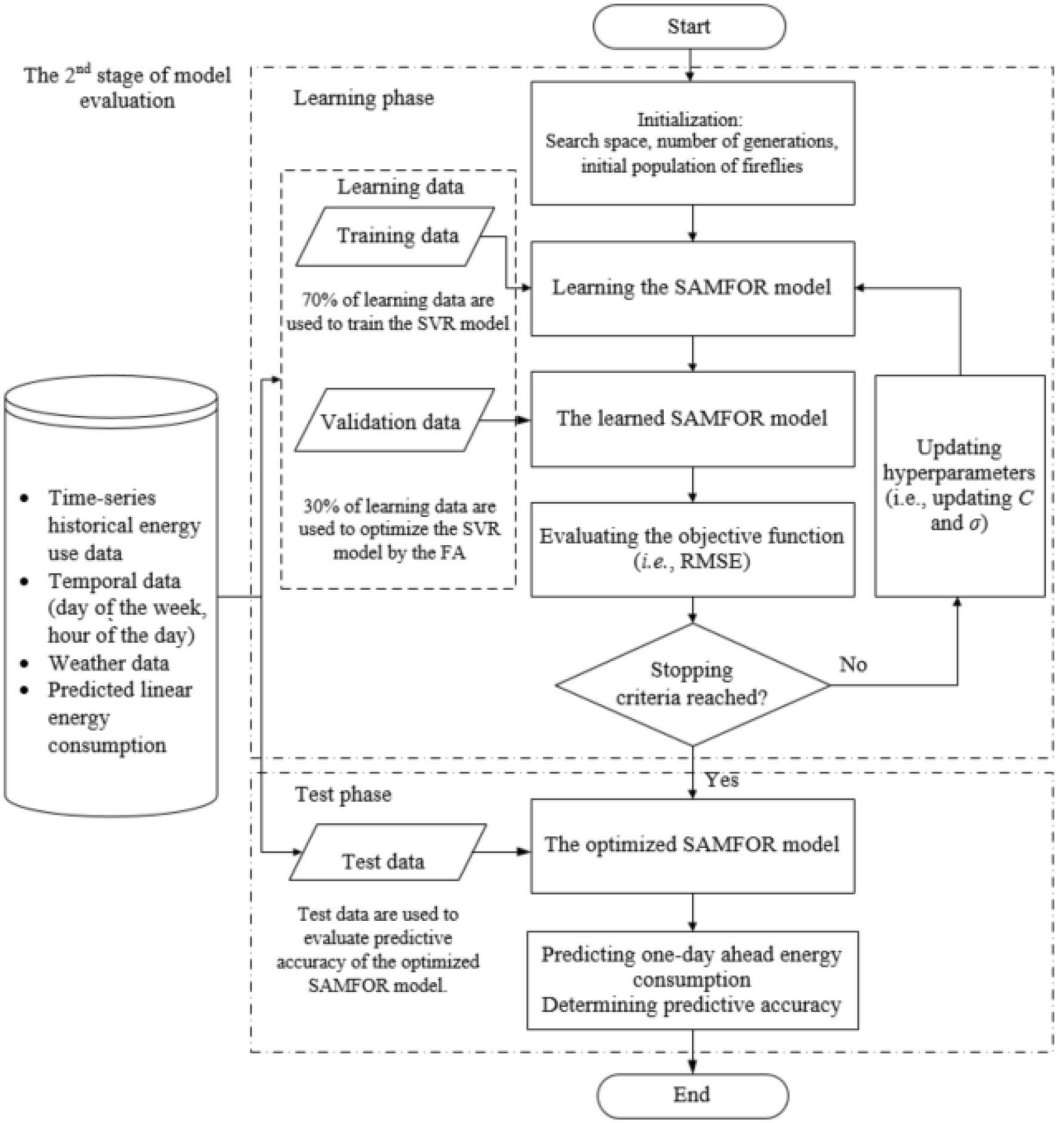
Implementation of the SAMFOR in the second stage.

The nonlinear time-series prediction model was built based on the integration of the SVR model and the FA optimization algorithm (FA-SVR). The FA was integrated to optimize hyperparameters of the SVR model. This integration can significantly improve the predictive performance of the proposed model because the configuration of the SVR model was optimized automatically to fit with data patterns. Figure 4 presents the implementation of the SAMFOR model in the 2nd stage.

The SARIMA projected the predicted linear building energy consumption in the 1st stage based on the learning data (LD). LD. In the 2nd stage, the proportion of LD (i.e., 70% of the total size of the learning data) was applied to train the SAMFOR model while the remaining proportion of the LD (i.e., 30%) was used to optimize the predictive accuracy of the proposed model via the optimization process by the FA. The FA determined the optimal hyperparameters of the SAMFOR in the search space via the objective function (OF). In this study, the root-mean-square error (RMSE) was used as the OF for the optimization problem. The RMSE is a statistical index that was calculated upon the collected actual building energy consumption data and predicted building energy consumption data. The operation of the FA was described in section “Firefly algorithm”. After the learning phase, the learned p model was produced. The accuracy of the learned model was then tested in the test phase. The test data include the 24-h building energy consumption data. The SAMFOR model was performed in the MATLAB environment which is a programming and numeric computing platform.

To provide users with a reliable prediction model, the proposed model was experienced the learning phase and test phase using various data sets from real-world buildings. Particularly, the proposed model was learned and tested multiple times. During an evaluation, the learning data were to build the time-series prediction model for building energy consumption in the learning phase. In this study, the suitable size of the learning data will be determined by the sensitivity analysis with different scenarios as stated in section “Sensitivity analysis”.

### Accuracy measures

The correlation coefficient (R), root-mean-square-error (RMSE), mean absolute error (MAE), and mean absolute percentage error (MAPE) measures were calculated based on Eqs. (7)–(10) to access the predictive accuracy of the SAMFOR model.

7 $${\text{R}} = \frac{{n\sum {y_{i} .y_{i}^{\prime } - \left( {\sum {y_{i} } } \right)\left( {\sum {y_{i}^{\prime } } } \right)} }}{{\sqrt {n\left( {\sum {y_{i}^{2} } } \right) - \left( {\sum {y_{i} } } \right)^{2} } \sqrt {n\left( {\sum {y_{i}^{\prime 2} } } \right) - \left( {\sum {y_{i}^{\prime } } } \right)^{2} } }}$$

8 $${\text{RMSE}} = \sqrt {\frac{1}{n}\sum\limits_{i = 1}^{n} {\left( {y_{i} - y_{i}^{\prime } } \right)^{2} } }$$

9 $${\text{MAE}} = \frac{1}{n}\sum\limits_{i = 1}^{n} {\left| {y_{i} - y_{i}^{\prime } } \right|}$$

10 $${\text{MAPE}} = \frac{1}{n}\sum\limits_{i = 1}^{n} {\left| {\frac{{y_{i} - y_{i}^{\prime } }}{{y_{i} }}} \right|}$$

where $y^{\prime}$ is predicted energy consumption, *y* is te actual energy consumption, and *n* is size of the data sample.

## Data collection and analytical results

### Energy consumption data and weather data

This study collected building energy consumption data from six buildings in Danang city in Vietnam. Danang is Vietnam's third-largest city in Vietnam and is located in Central Vietnam. Energy consumption data were collected within two years of 2018 and 2019 in the 30-min resolution. Energy unit prices in Vietnam can be expected to vary along with the time of consuming energy. Thus, future prediction results of energy consumption can be used as a reference to shift and optimize the operating time of appliances, lighting, and an air conditioning system in buildings. The 30-min interval of data collection was selected with respect to optimization of building operational schedule in the future.

Besides, energy consumption data from buildings in Danang city was automatically collected by smart meters namely Automatic Meter Reading (AMR). This AMR system is capable of transmitting collected data to Operator Center every 30 min. Whenever enterprises receive notifications from the Load Dispatch Centre, they can reduce electricity consumption within 30 min. Short-term forecasting, e.g., one-day ahead, is of prime importance in day-to-day market operations. Thus, exactly forecasting electric consumption every 30 min will support both consumers and the power industry saving energy consumption. There were 35,040 data points for each building in 2 years. This was a large dataset that was used to evaluate the proposed prediction model in this study.

**Figure 5:**
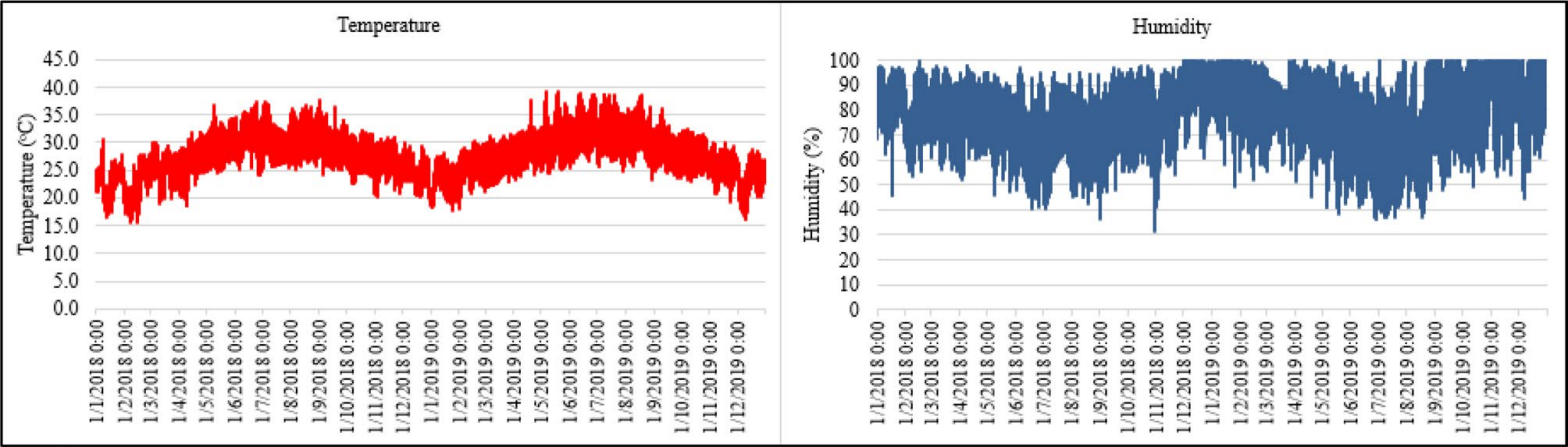
Weather data in the years of 2018 and 2019.

For improving the effectiveness of the prediction model, the weather data in Danang city was also recorded for two years of 2018 and 2019 in the 30-min resolution. Danang is belonging in the zone of typical tropical monsoon, temperate and equable climate.Figure 5 visualized the temperature and humidity data collected in 2018 and 2019.

**Figure 6:**
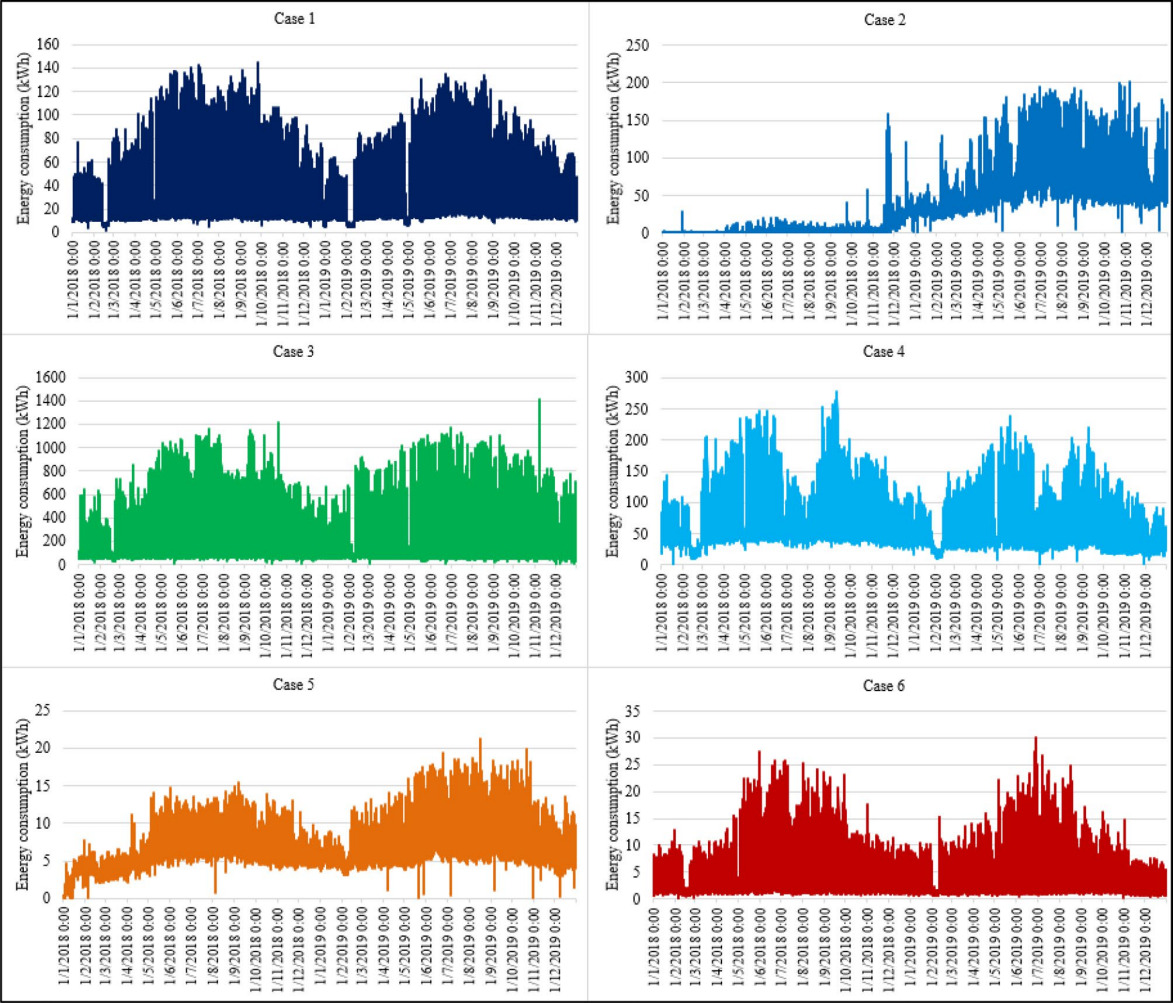
Energy consumption in the buildings in the years of 2018 and 2019.

**Figure 7:**
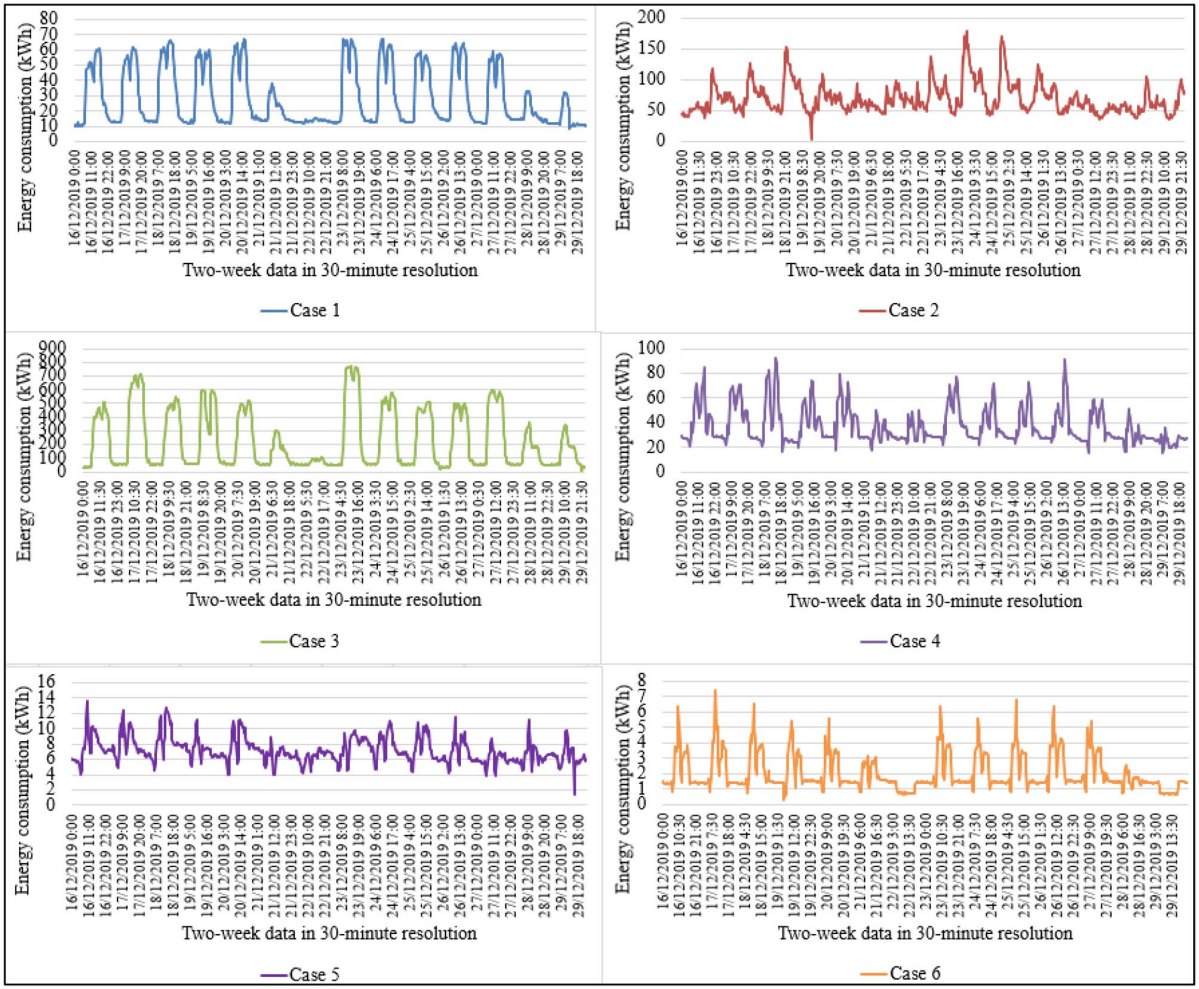
Energy use profile in a week (16th–29th December 2019).

For demonstrating the applicability of the proposed SAMFOR model, energy consumption data in six buildings was used. These datasets were selected randomly from the entire datasets. They are various types of building including office buildings, hotels, administration buildings, and educational buildings. They were in various locations in Danang city and have different operational characteristics. Table 1 presents their information including the building identification, the management identification, the station identification, the customer code, the locations, and their building type. Their energy use profiles for the years of 2018 and 2019 were plotted in Fig. 6, which reveal the complex patterns and highly random energy consumption among buildings. Figure 7 provides readers with two-week energy consumption profiles in six buildings.

**Table 1:** Buildings in the database.

Case	Building ID	Building type	Management ID	Station ID	Customer code	Location / district
1	80	Office building	PP0100	AD53ABCQ	PP01000133789	Hai Chau
2	48	Hotel	PP0100	AD53A9BB	PP01000646015	Hai Chau
3	144	Administration building	PP0100	AD53ABGY	PP01000639871	Hai Chau
4	179	Educational building	PP0300	HD53HAAK	PP03000800027	Lien Chieu
5	540	Office building	PP0700	VD53VACX	PP07000600463	Cam Le
6	547	Office building	PP0700	VD53VAFG	PP07000673738	Cam Le

### Sensitivity analysis

Sensitivity analyses were performed to select appropriate inputs for the prediction. Table 2 presents the descriptive statistics of data attributes that may impact energy use in buildings. They consist of the historical energy consumption in 30-min intervals (*Y*), the outdoor temperature (*T*), the outdoor humidity (*H*), day of the week (*DoW*), and hour of the day (*HoD*). Identifying suitable inputs for the prediction was meaningful to achieving reliable and accurate prediction results.

**Table 2:** Data description of case studies.

Case	30-minutely energy consumption *Y* (kWh)				Outdoor temperature *T* (^o^C)				Outdoor humidity *H* (%)				Day of the week—DoW	Hour of the day—HoD
	Min	Ave	Max	Std. dev	Min	Ave	Max	Std. dev	Min	Ave	Max	Std. dev		
1	1.74	35.08	144.79	31.49	15.5	27.1	39.3	3.9	31.0	79	100	14	Mon.,	0,
2	0.04	37.40	201.03	42.74	15.5	27.1	39.3	3.9	31.0	79	100	14	Tue.,	1,
3	2.11	273.32	1411.52	292.01	15.5	27.1	39.3	3.9	31.0	79	100	14	Wed.,	2,
4	0.02	62.94	277.62	38.69	15.5	27.1	39.3	3.9	31.0	79	100	14	Thur.,	…,
5	0.00	7.25	21.23	2.63	15.5	27.1	39.3	3.9	31.0	79	100	14	Fri.,	22,
6	0.06	4.15	30.10	4.30	15.5	27.1	39.3	3.9	31.0	79	100	14	Sat., Sun	23

The prediction accuracy of models depends on (1) input parameters such as outdoor temperature, outdoor humidity, historical energy consumption patterns, and temporal data; (2) length size of the learning data used to train models; and (3) lag values as mentioned in section “Learning and test process of the proposed SAMFOR model”. Therefore, three sensitivity analyses were performed in this study to configure prediction models as summarized in Table 3. The 1st sensitivity analysis aims to select an appropriate value of lag for time-series energy use prediction in buildings. There are 48 scenarios in which the lag value varies from 1 to 48 (*i.e.*, lag = 1, 2, 3, …., 48). The 2nd sensitivity analysis aims to select an appropriate set of inputs for time-series energy use prediction in buildings. There are 6 scenarios considering the impact of a different combination of inputs among temporal data, weather data, and historical energy consumption data. The 3rd sensitivity analysis aims to select an appropriate size of the learning data for time-series energy use prediction in buildings. There are 13 scenarios with different lengths of learning data. The size of the learning data varies from 3 to 15 months. 67 simulations were performed in three sensitivity analyses.

**Table 3:** Summary of three sensitivity analyses.

Sensitivity analysis	Scenario	Description/aims
1. Lag selection	48 scenarios: 1 → 48	There are 48 scenarios in which the lag value varies from 1 to 48 (*i.e.*, lag = 1, 2, 3, …., 48)
2. Inputs selection	Scenario 1	Scenario 1 considers only the historical building energy consumption *Y* as the input for the prediction
	Scenario 2	Inputs in scenario 2 are *Y*, *ToD,* and *DoW*
	Scenario 3	Inputs in scenario 3 are *Y* and *T*
	Scenario 4	Inputs in scenario 4 are *Y, T,* and *H*
	Scenario 5	Inputs in scenario 5 are *Y*, *T*, *ToD*, and *DoW*
	Scenario 6	Inputs in scenario 6 are *Y*, *T*, *H*, *ToD*, and *DoW*
3. Size selection of learning data	13 scenarios 1 → 13	There are 13 scenarios with different lengths of the learning data. The size of the learning data varies from 3 to 15 months

**Figure 8:**
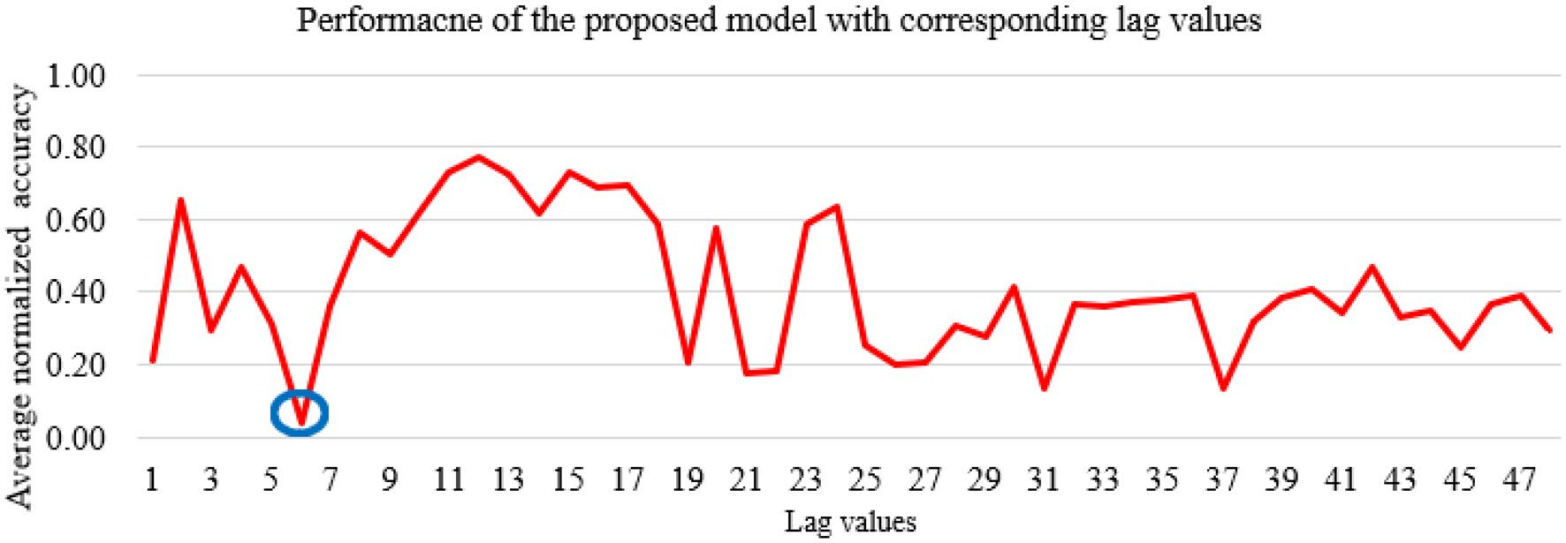
Average normalized predictive accuracy with different lag values.

Four measures including R, RMSE, MAE, and MAPE were used to compare the predictive accuracy of the SAMFOR model with different values of the lag. Figure 8 depicts the average normalized predictive accuracy with different lag values in the 1st sensitivity analysis. The comparisons show that the proposed SAMFOR model obtained the best accuracy with a lag value of 6. Thus, the lag of 6 was set in the SAMFOR model for predicting future energy use in buildings.

In the 2nd sensitivity analysis, the results were presented in Table 4. Using the different combinations of input resulted in a difference accuracy. The combination of the historical energy consumption, outdoor temperature, and temporal data as inputs in Scenario 5 has the best accuracy among the six scenarios. Therefore, this input set was applied in predicting one-day-head energy consumption in buildings.

**Table 4:** Sensitivity analysis 2 for identifying the appropriate inputs.

Scenario	Combinations of different inputs for predicting energy consumption	Accuracy indices with the test data				ANC	Rank
		RMSE (kWh)	MAE (kWh)	MAPE (%)	R		
1	*Y*	1.24	1.53	4.69	0.991	0.88	6
2	*Y*, *HoD,* and *DoW*	1.18	1.40	4.38	0.993	0.70	5
3	*Y* and *T*	1.15	1.32	4.18	0.993	0.47	3
4	*Y, T,* and *H*	1.16	1.34	4.46	0.993	0.53	4
5	*Y*, *T*, *HoD*, and *DoW*	**1.07**	**1.14**	**3.42**	**0.995**	**0.00**	**1**
6	*Y*, *T*, *H*, *HoD*, and *DoW*	1.08	1.16	3.53	0.995	0.06	2

Note: ANC stands for average normalized accuracy; A scenario with bold numbers indicate its outstanding performance compared to others.

Selecting an appropriate size of the learning data is essential to reduce computational effort and improve predictive accuracy. The 3rd sensitivity analysis aims to select an appropriate size of the learning data. Accuracy indices of the proposed SAMFOR model were assessed by varying the learning data from 3 to 15 months. The longer the learning data is, the higher the computational effort is needed. For example, the computational time was about 1 min for one evaluation of the model as the 3-month learning data were applied, while it was about 1 h as the 15-month learning data were applied. The results in Table 5 revealed that the MAPE values were less than 5% and the R values were greater than 0.990 for all scenarios. The model performance did not vary much along with the change of the learning data for the short-term energy use prediction. The comparison results confirmed that the model was the best in prediction when the learning data is four months. Therefore, 4-month historical energy use data were set for predictions.

**Table 5:** Sensitivity analysis 3 for identifying the appropriate learning data size.

Scenario	Learning data size (months)	Accuracy indices with the test data				ANC	Rank
		RMSE (kWh)	MAE (kWh)	MAPE (%)	R		
1	3	1.23	1.52	4.65	0.991	0.485	8
2	4	**1.21**	**1.46**	**4.40**	**0.992**	**0.091**	**1**
3	5	1.23	1.52	4.59	0.991	0.296	3
4	6	1.24	1.54	4.68	0.991	0.389	7
5	7	1.24	1.53	4.64	0.991	0.369	6
6	8	1.26	1.59	4.75	0.991	0.557	10
7	9	1.27	1.62	4.83	0.990	0.730	12
8	10	1.28	1.64	4.89	0.990	0.819	13
9	11	1.25	1.56	4.79	0.991	0.629	11
10	12	1.25	1.55	4.72	0.991	0.494	9
11	13	1.23	1.50	4.57	0.991	0.326	4
12	14	1.22	1.50	4.54	0.991	0.333	5
13	15	1.20	1.45	4.50	0.992	0.242	2

Significant values are in [bold].

### Analytical results and discussion

Table 6 presents the model settings for one-day-ahead building energy consumption in a 30-min resolution. These settings were based on the three above sensitivity analyses. The SARIMA model was set as SARIMA (1*, 0, 1*) × (48*,* 0, 48)_48_. The seasonal length was set as 48 which consists of a recorded number of data points in a day. The search space for C and *σ* were set in the range of [10^−3^ 10^12^]. These hyperparameters were optimized by the FA throughout minimizing the objective function of the root-mean-square error. The firefly’s population and maximum iteration were set at 50 and 25, respectively.

**Table 6:** Model settings for prediction.

Parameter	Setting
SARIMA	SARIMA (1, 0, 1) × (48*, 0, 48*)_48_
FA-SVR	*C* ~ [10^−3^ 10^12^]; *σ* ~ [10^−3^ 10^12^]; Population = 50; Maximum iteration = 25; *β*_min_ = 0.1; *γ* = 1
The learning data	4-month historical building energy consumption data in 30-min resolution
Inputs	*Y*, *T*, *ToD*, and *DoW*
Lag	6

For evaluating the performance of the SAMFOR model, data from six buildings were used. Table 7 presents data divisions that were used for twenty-four evaluations in which four evaluations were performed for each building. Twenty-four evaluations were performed to test the predictive accuracy of the SAMFOR model. Data were divided into the learning data and test data. For example, in Table 7, the learning data were from April 1—July 31, 2019, while test data were on August 1, 2019, for each building. The learning size was 4-month data which is the result of the 3rd sensitivity analysis in section “Sensitivity analysis”. As mentioned in section “Sensitivity analysis”, this learning size was the best choice for learning the proposed model that can achieve the best prediction accuracy. Besides, in this study, the length of prediction of energy consumption was a 48-step-ahead prediction. The size of test data was aligned with the length of prediction of energy consumption to evaluate how the effectiveness of the prediction model.

**Table 7:** Data settings for evaluations of all buildings.

Evaluation	Period of learning data		Period of test data
	4 months	Sample size (points)	
1	January 8–May 7, 2018	5696	May 8, 2018 (Tuesday)
2	June 15–October 14, 2018	5856	October 15, 2018 (Monday)
3	April 1–July 31, 2019	5856	August 1, 2019 (Thursday)
4	August 27–December 26, 2019	5856	December 27, 2019 (Friday)

Table 8 presents performance results obtained by the proposed SAMFOR model via statistical indices of RMSE, MAE, MAPE, and R during the learning phase and test phase. Each building was evaluated four times to ensure generalizability. The average accuracy measures across twenty-four evaluations in six buildings were 1.77 kWh in the root-mean-square-error, 5.02 kWh in the mean absolute error, 9.56% in the mean absolute percentage error, and 0.914 in the correlation coefficient over the test phase. For all buildings, the obtained R values were greater than 0.900 which shows a great agreement between actual data and prediction data by the proposed SAMFOR hybrid time-series model.

**Table 8:** Performance results by SAMFOR model for six buildings in the learning phase and test phase.

Building	Evaluation	Performance by SAMFOR in learning phase				Performance by SAMFOR in test phase			
		RMSE (kWh)	MAE (kWh)	MAPE (%)	R	RMSE (kWh)	MAE (kWh)	MAPE (%)	R
Building 1	1	1.24	1.54	6.77	0.993	1.69	2.86	5.95	0.996
	2	1.54	2.36	6.63	0.995	1.40	1.97	5.20	0.996
	3	1.39	1.94	5.76	0.996	1.44	2.08	4.45	0.996
	4	1.27	1.61	5.22	0.995	1.11	1.23	3.65	0.995
	Average	1.36	1.86	6.10	0.995	1.41	2.04	4.81	0.996
Building 2	1	0.37	0.14	8.16	0.915	0.70	0.49	11.33	0.899
	2	0.69	0.48	15.94	0.917	0.61	0.38	13.51	0.951
	3	2.66	7.10	9.95	0.956	2.44	5.97	8.36	0.959
	4	2.56	6.56	9.93	0.956	2.15	4.60	8.75	0.843
	Average	1.57	3.57	10.99	0.936	1.48	2.86	10.49	0.913
Building 3	1	3.89	15.15	10.53	0.989	5.12	26.23	13.84	0.992
	2	4.43	19.65	12.68	0.992	3.98	15.81	18.89	0.842
	3	4.36	18.99	12.40	0.994	4.63	21.46	8.26	0.994
	4	4.08	16.68	10.74	0.993	4.23	17.94	9.76	0.989
	Average	4.19	17.62	11.59	0.992	4.49	20.36	12.69	0.954
Building 4	1	2.06	4.25	25.72	0.982	2.49	6.20	6.25	0.991
	2	2.16	4.67	6.43	0.984	1.91	3.66	6.90	0.868
	3	2.17	4.70	9.31	0.985	1.77	3.13	7.07	0.865
	4	1.91	3.65	8.91	0.983	1.71	2.94	8.02	0.890
	Average	2.08	4.32	12.59	0.983	1.97	3.98	7.06	0.904
Building 5	1	0.51	0.26	24.19	0.960	0.71	0.50	6.43	0.922
	2	0.64	0.41	5.17	0.933	0.55	0.30	4.87	0.904
	3	0.71	0.50	8.48	0.953	0.71	0.50	7.25	0.782
	4	0.70	0.49	7.09	0.958	0.48	0.23	4.19	0.955
	Average	0.64	0.42	11.23	0.951	0.61	0.38	5.69	0.891
Building 6	1	0.59	0.34	13.73	0.975	0.88	0.77	14.71	0.986
	2	0.76	0.58	13.57	0.983	0.54	0.29	28.92	0.407
	3	0.74	0.55	14.03	0.983	0.79	0.62	13.19	0.966
	4	0.57	0.33	12.71	0.975	0.47	0.22	9.65	0.946
	Average	0.67	0.45	13.51	0.979	0.67	0.48	16.62	0.827
Overall average		1.75	4.71	11.00	0.973	1.77	5.02	9.56	0.914
Standard deviation		1.31	6.28	5.27	0.024	1.40	7.41	5.63	0.124

**Figure 9:**
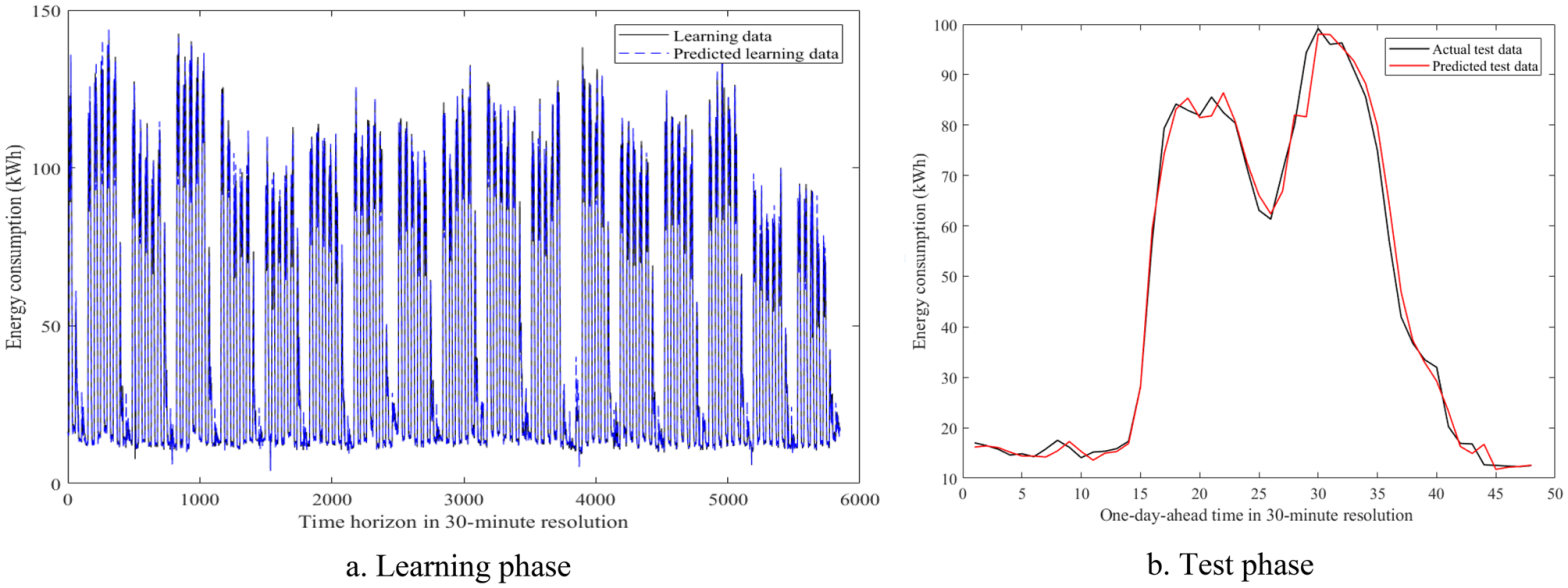
Actual and forecasted building energy profile in building 1 at 2nd evaluation.

**Figure 10:**
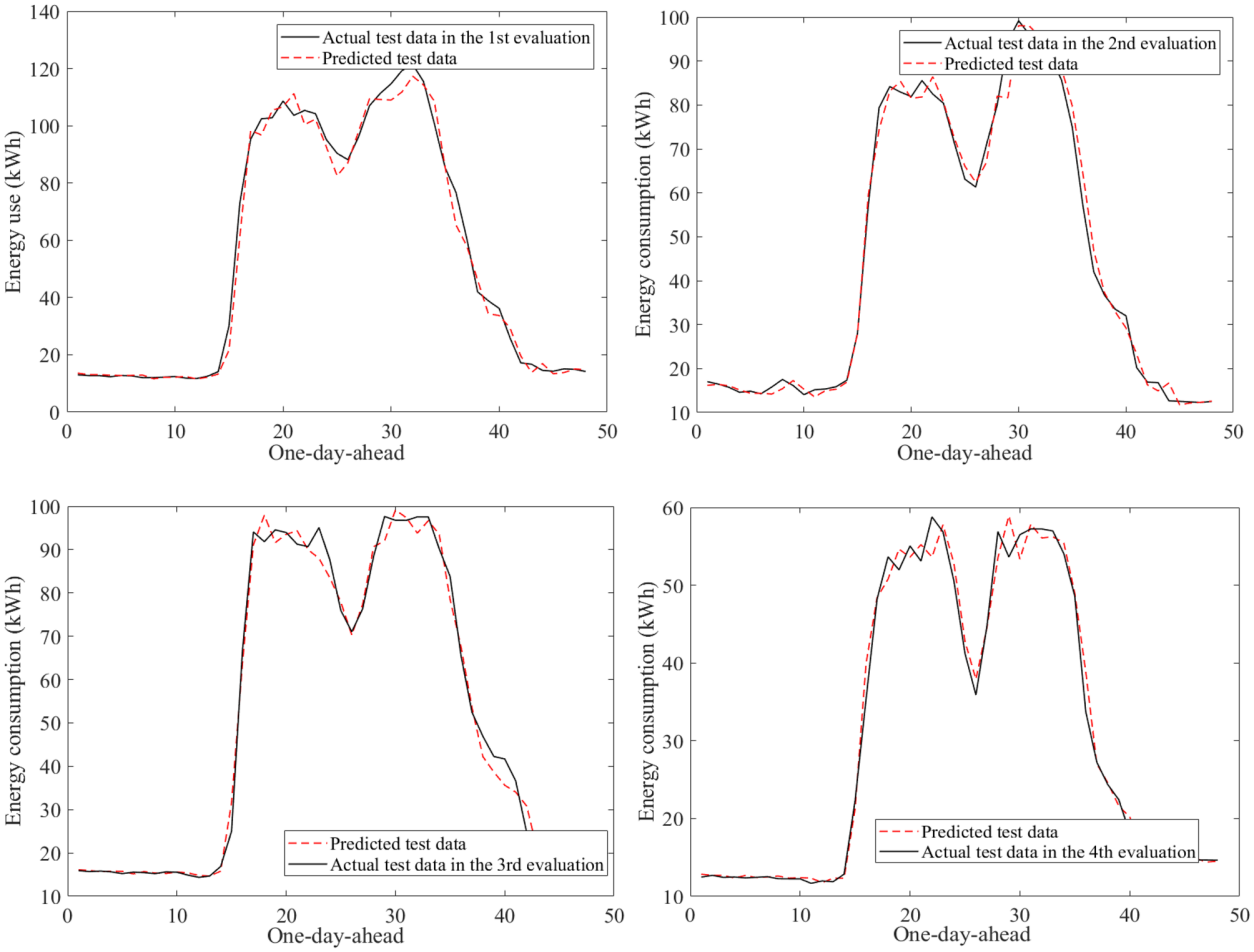
Actual and forecasted test data by the proposed SAMFOR model for building 1.

**Figure 11:**
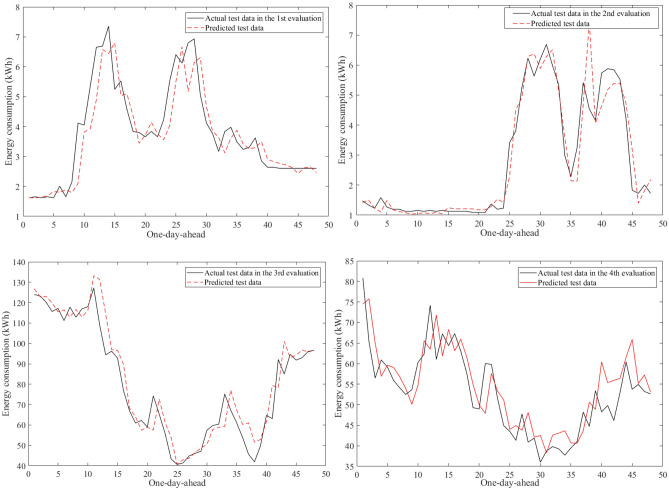
Actual and forecasted test data by the proposed SAMFOR model for building 2.

**Figure 12:**
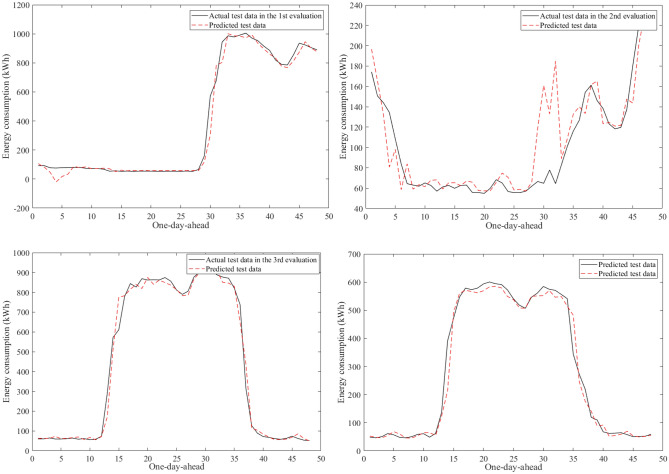
Actual and forecasted test data by the proposed SAMFOR model for building 3.

**Figure 13:**
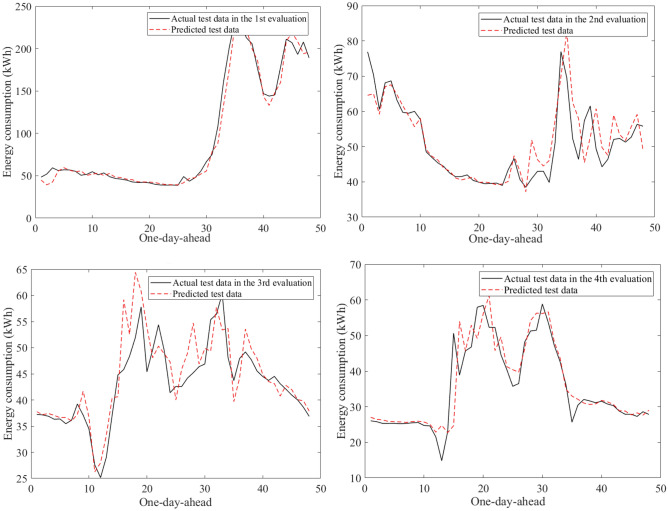
Actual and forecasted test data by the proposed SAMFOR model for building 4.

**Figure 14:**
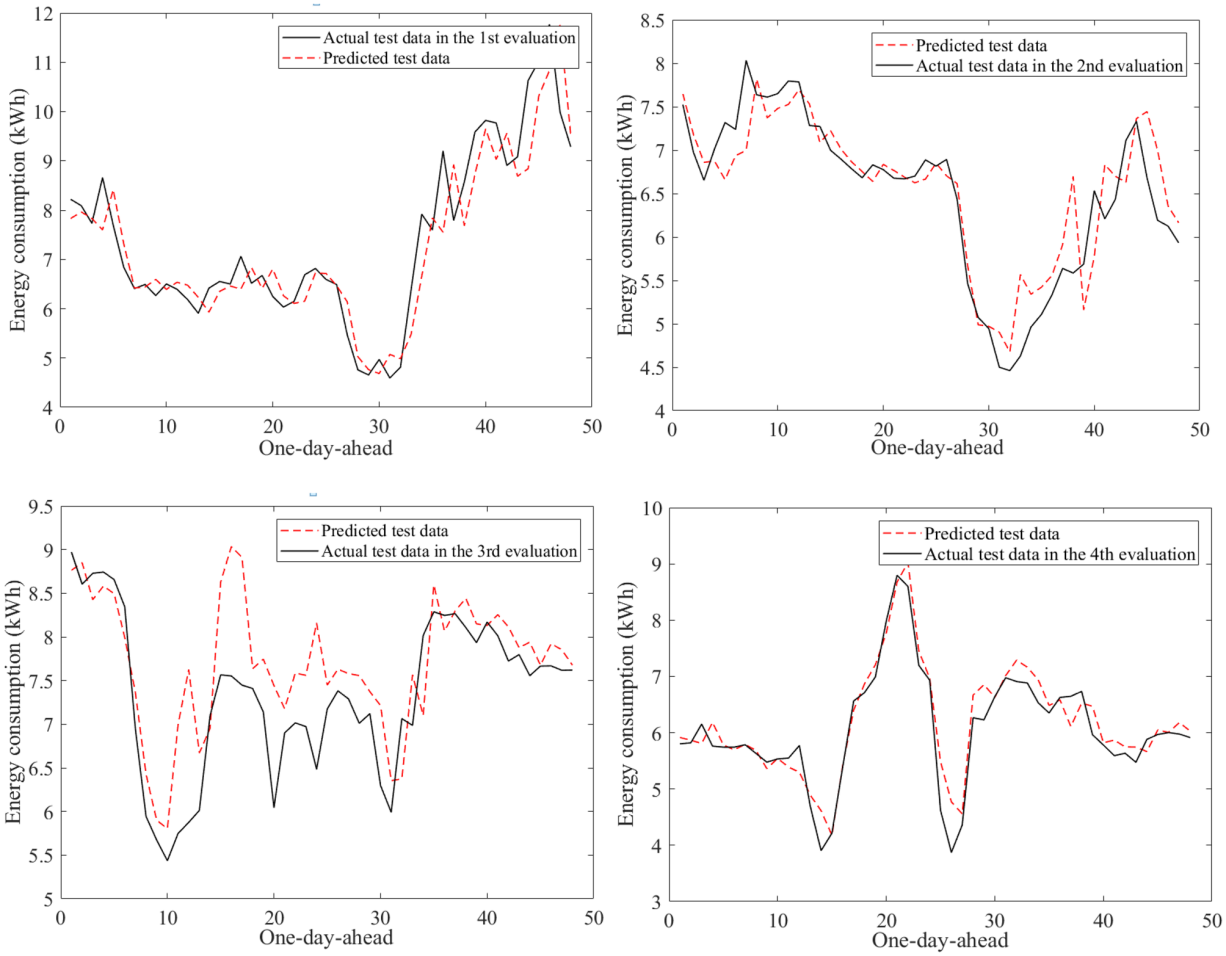
Actual and forecasted test data by the proposed SAMFOR model for building 5.

**Figure 15:**
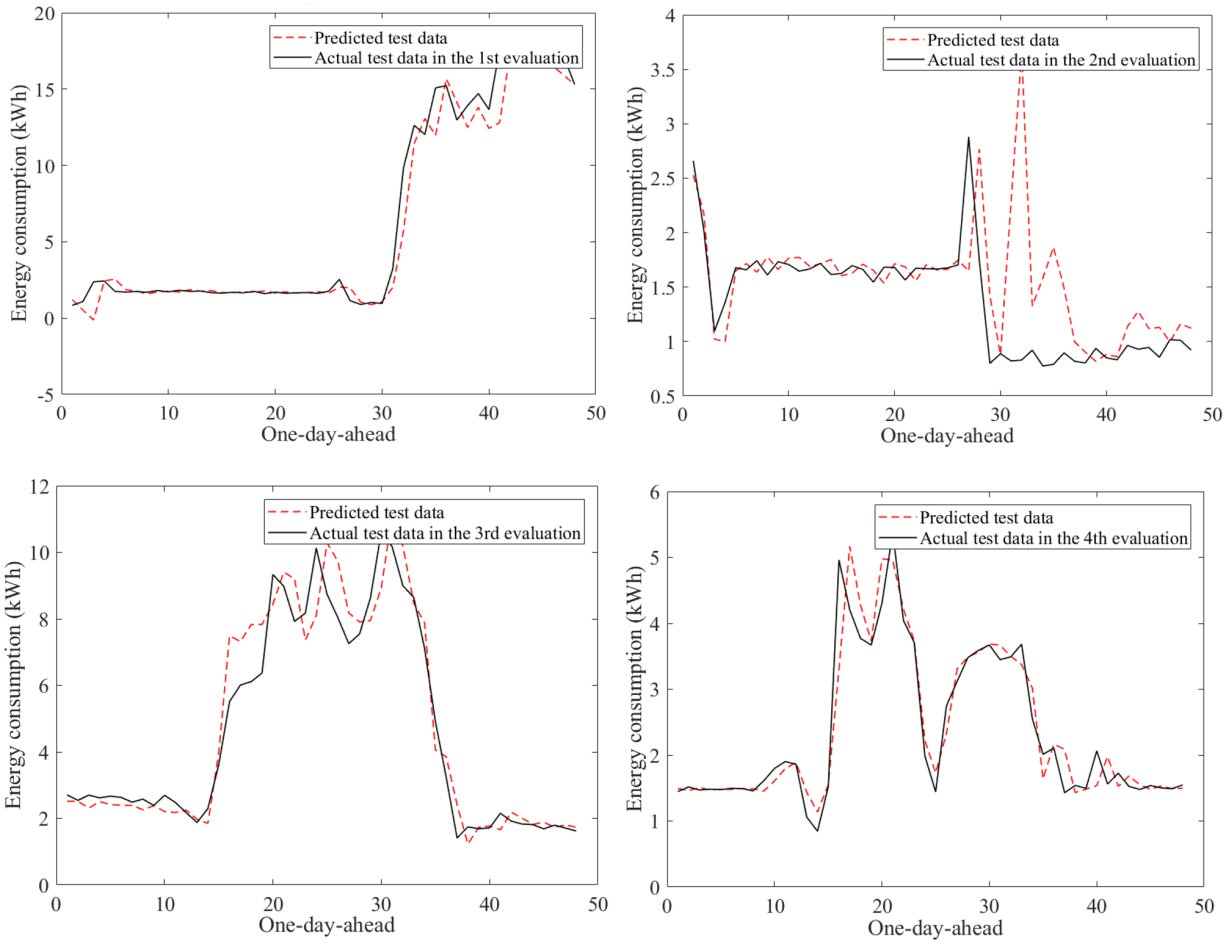
Actual and forecasted test data by the proposed SAMFOR model for building 6.

Figure 9 visualizes an example of the 30-min building energy consumption data between actual and forecasted data in building 1 at the second evaluation. They were very close to each other in the learning and test phases. The hybrid AI model achieved very low MAPE values through twenty-four evaluations in Table 8. The average MAPE values were about 4.81% for building 1, 10.49% for building 2, 12.69% for building 3, 7.06% for building 4, 5.69% for building 5, 16.62% for building 6. The comparisons of the prediction results produced by the SAMFOR model with actual data for six buildings in the test phase were visualized in Figs. 10, 11, 12, 13, 14, and 15. The visualization in these Figures revealed the high agreement between predicted and actual data on energy consumption. The prediction results confirmed the effectiveness of the SAMFOR in forecasting the short-term energy use profiles in buildings.

The optimal values of hyperparameters in the hybrid SAMFOR model and the computing time of the model were presented in Table 9. Most optimal *C* and *δ* were less than 1000. This suggests future settings can be narrowed within 1000 to save the computational cost in terms of computing memory and elapsed CPU time. The model was implemented in a computer with the processor of “Intel (R) Core (TM) i7-9750H CPU @ 2.60 GHz 2.59 GHz”, the RAM of 8.00 GB, the system type of 64-bit operating system, × 64-based processor. The elapsed CPU time was about 4 min for four evaluations which means a minute per evaluation. The results reveal the efficiency of the proposed model which is very meaningful for the short-term energy prediction.

**Table 9:** Optimal hyperparameters of SAMFOR model and computing time.

Building	Eval	*C*	*δ*	Elapsed CPU time (min)	Building	Eval	*C*	*δ*	Elapsed CPU time (min)
1	1	946.32	203.20	4.24	4	1	856.43	491.83	3.62
	2	967.85	124.48			2	860.90	479.01	
	3	982.64	68.22			3	2.79 × 10^10^	3.55 × 10^9^	
	4	965.27	134.09			4	42.57	163.03	
2	1	1.46 × 10^9^	7.01 × 10^7^	3.64	5	1	4.60 × 10^7^	2.89 × 10^9^	3.65
	2	16.93	66.58			2	1.02 × 10^9^	2.51 × 10^7^	
	3	1.34 × 10^9^	4.08 × 10^10^			3	948.92	193.87	
	4	175.34	578.39			4	416.84	972.34	
3	1	2.89 × 10^10^	6.16 × 10^9^	3.85	6	1	877.76	429.19	4.15
	2	961.69	147.36			2	956.51	166.40	
	3	969.42	118.57			3	950.85	186.94	
	4	978.21	85.25			4	946.10	204.00	

The performance of the proposed SAMFOR model was compared to those of the linear time-series prediction model (i.e., SARIMA) and base nonlinear time-series prediction models (i.e., support vector regression (SVR), random forests (RF), and an integration of SARIMA and SVR model. These models have been widely applied for building energy consumption. Table 10 reveals performance by the baseline SVR and RF models. The SARIMA model was not stable in short-term building energy consumption prediction. It performed quite well for buildings 1 and 5 in which the obtained MAPE values were lower than 15%. For the remaining cases, the SARIMA model showed poor results with the high MAPE of from 30.44% up to 129.30%. The reason behind this poor result is that the SARIMA model assumed the linear relationship between future data and historical data. The inherent assumption limited its power in modeling the complex and nonlinear patterns in building energy use data. The SVR and RF models were more stable than the SARIMA model in the prediction. The overall average MAPE values were 9.89% by the SVR model and 10.18% by the RF model through twenty-four evaluations in six buildings. These good results depict the flexibility of the SVR and RF models in capturing the highly-vary patterns in energy data.

**Table 10:** Predictive performance by SVR and RF models.

Building	Evaluation	Performance by SVR				Performance by RF			
		RMSE (kWh)	MAE (kWh)	MAPE (%)	R	RMSE (kWh)	MAE (kWh)	MAPE (%)	R
Building 1	1	0.86	0.60	13.47	0.868	0.82	0.57	14.65	0.871
	2	5.90	3.84	8.48	0.984	3.94	2.58	6.50	0.993
	3	7.17	3.79	6.59	0.979	3.96	2.58	5.42	0.994
	4	4.12	2.50	6.82	0.976	2.83	1.84	5.36	0.989
	Average	4.51	2.68	8.84	0.952	2.89	1.89	7.98	0.961
Building 2	1	0.86	0.60	13.47	0.868	0.82	0.57	14.65	0.871
	2	0.87	0.52	17.09	0.901	0.65	0.42	16.43	0.945
	3	8.19	6.01	8.49	0.956	9.31	7.30	10.39	0.946
	4	5.47	4.29	7.95	0.850	6.12	4.87	9.19	0.818
	Average	3.85	2.86	11.75	0.894	4.22	3.29	12.67	0.895
Building 3	1	71.27	25.50	7.02	0.986	48.06	22.25	8.00	0.994
	2	12.38	8.71	8.56	0.967	18.80	12.08	11.92	0.925
	3	73.59	35.10	11.7	0.981	38.45	21.82	8.49	0.995
	4	50.49	23.31	11.62	0.978	40.83	23.36	10.88	0.986
	Average	51.94	23.15	9.73	0.978	36.53	19.88	9.82	0.975
Building 4	1	16.44	9.30	7.87	0.978	11.74	7.36	6.14	0.988
	2	6.76	4.39	8.01	0.796	7.25	4.49	8.38	0.767
	3	4.85	3.13	7.38	0.861	5.11	3.25	7.63	0.817
	4	5.90	3.57	9.73	0.882	5.17	3.06	8.27	0.907
	Average	8.49	5.10	8.25	0.879	7.32	4.54	7.61	0.870
Building 5	1	0.74	0.55	7.17	0.908	0.83	0.58	7.35	0.902
	2	0.34	0.25	3.93	0.933	0.44	0.33	5.46	0.893
	3	0.58	0.44	6.29	0.839	0.63	0.48	6.84	0.799
	4	0.63	0.39	6.95	0.807	0.62	0.42	7.33	0.819
	Average	0.57	0.41	6.09	0.872	0.63	0.45	6.75	0.853
Building 6	1	1.54	0.84	15.13	0.979	2.71	1.49	18.58	0.957
	2	0.33	0.18	13.68	0.773	0.53	0.29	23.12	0.621
	3	0.90	0.60	13.41	0.957	0.73	0.47	9.32	0.970
	4	0.71	0.40	16.49	0.801	0.55	0.33	14.00	0.897
	Average	0.87	0.50	14.68	0.877	1.13	0.65	16.26	0.861
Overall average		11.70	5.78	9.89	0.909	8.79	5.12	10.18	0.903
Standard deviation		21.36	9.14	3.60	0.071	13.79	7.30	4.58	0.093

**Figure 16:**
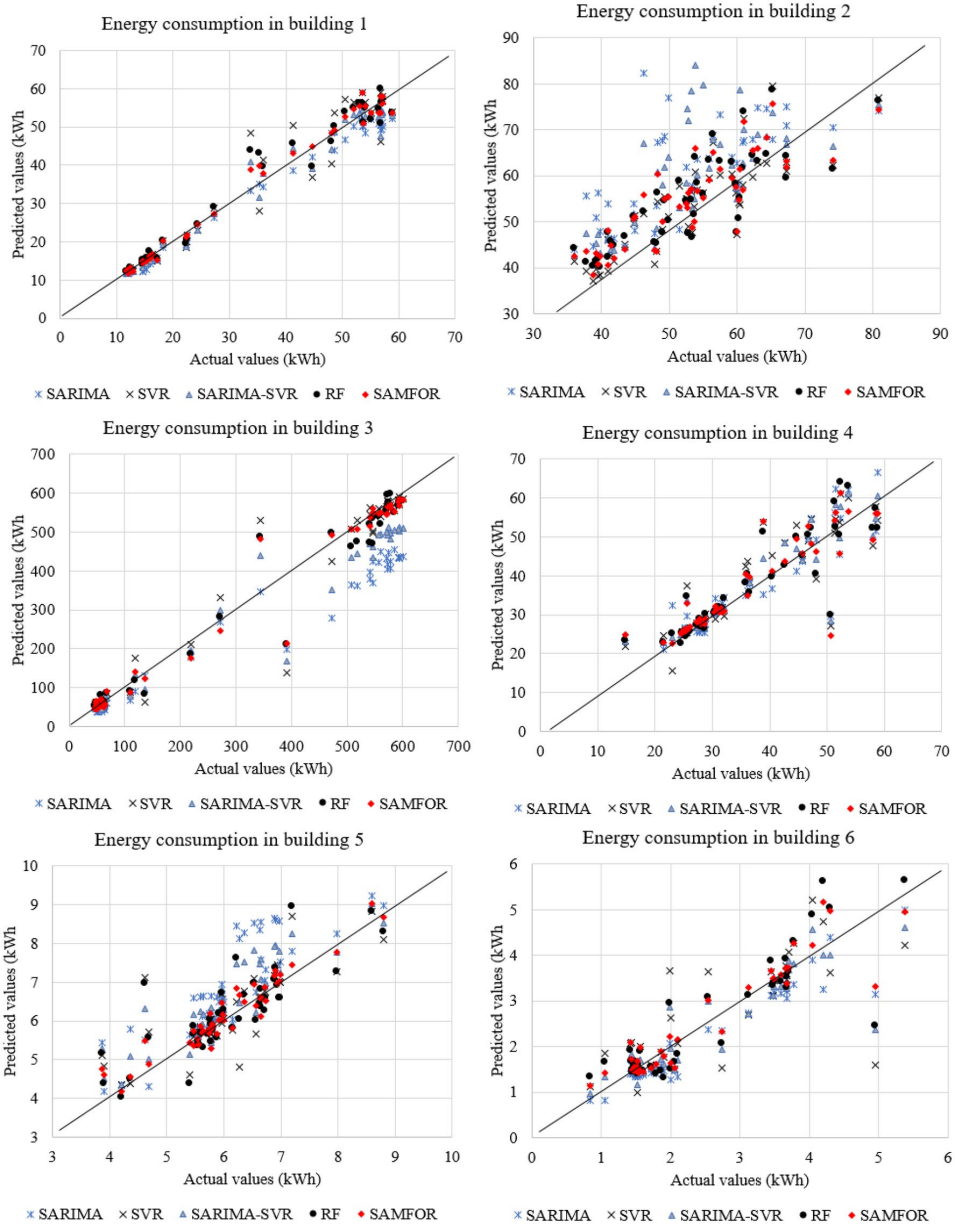
Comparison of prediction results by all investigaged models.

**Figure 17:**
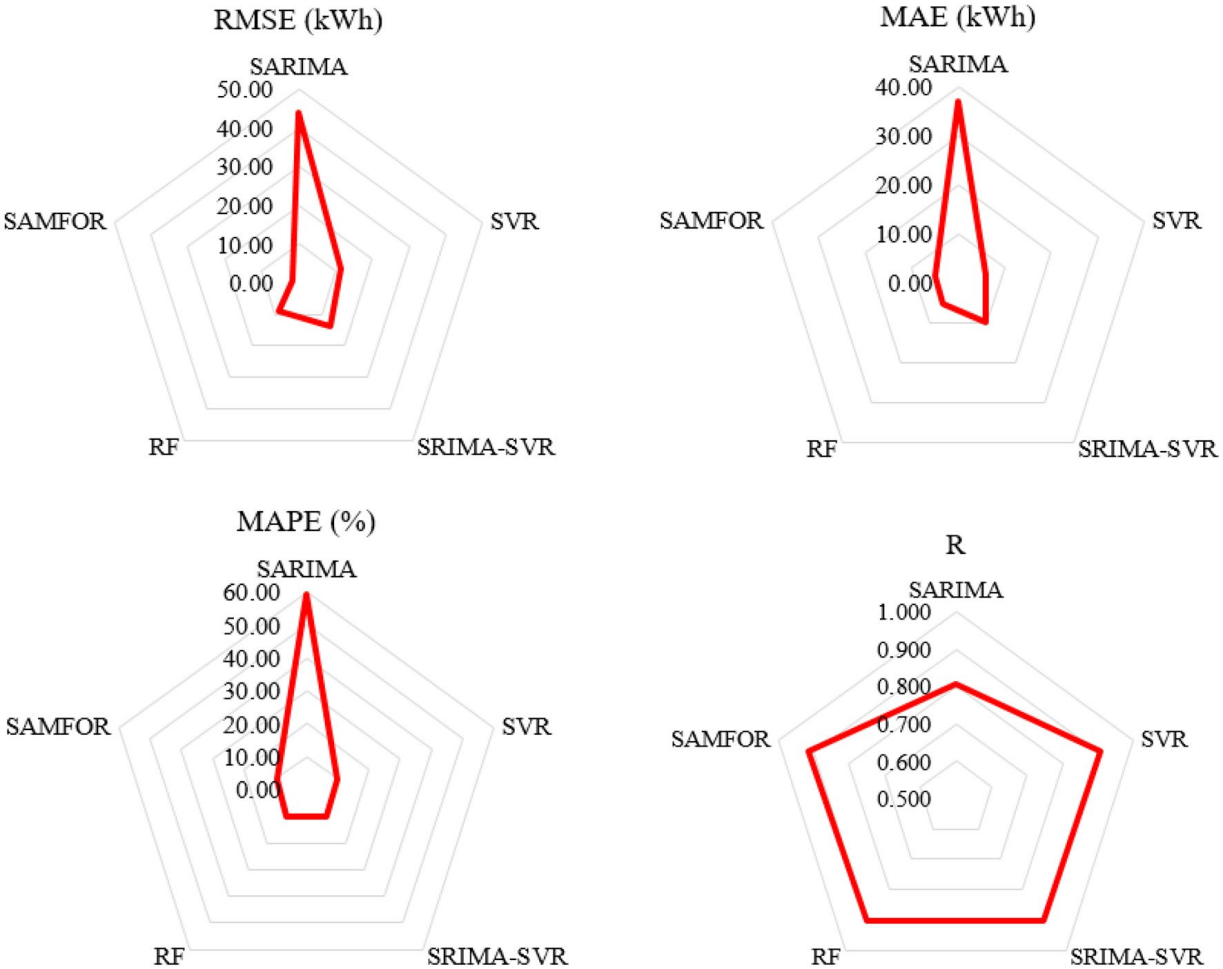
Performance comparison among models.

A performance comparison between the proposed SAMFOR model and SARIMA, SVR SARIMA-SVR, and RF models was presented in Table 11. Figure 16 shows comparisons of the one-day ahead prediction outputs among models in the scatter plots. Comparison results show that the SAMFOR model was more effective than the others in terms of all accuracy measures in forecasting short-term energy use in buildings. The proposed model has the lowest errors with 1.77 kWh in the RMSE, 5.02 kWh in the MAE, 9.56% in the MAPE, and 0.914 in the R. The hybrid artificial intelligence approach can improve the accuracy from 7.0 to 42.3 kWh in the RMSE as compared to baseline models. Figure 17 visualizes the comparison of the MAPE, MAE, RMSE, and R values obtained by the proposed SAMFOR, SVR, RF, SARIMA, and SARIMA-SVR models. The comparison revealed that the performance index of the proposed SAMFOR were better that those of other investigated models. The results confirmed the effectiveness of the SAMFOR model in predicting energy consumption in buildings.

**Table 11:** Performance comparison among base models and proposed model.

Model/method	Performance indices				Improvement by the SAMFOR			
	RMSE (kWh)	MAE (kWh)	MAPE (%)	R	RMSE (kWh)	MAE (kWh)	MAPE (%)	R
SARIMA	44.08	36.94	58.19	0.806	42.3	31.9	49.6	0.108
SVR	11.70	5.78	9.89	0.909	9.9	0.8	0.3	0.005
SRIMA-SVR	13.91	9.83	10.42	0.902	12.1	4.8	0.9	0.012
RF	8.79	5.12	10.18	0.903	7.0	0.1	0.6	0.011
Proposed SAMFOR	1.77	5.02	9.56	0.914

The accuracy of the M5P and random tree (RT) models yielded 23.88% and 40.73% in the MAPE, respectively^42^ while the SVR model in^39^ obtained 16.01% in MAPE in the day-ahead prediction of hourly energy use in buildings. The proposed SAMFOR model reached 9.56% in the MAPE in predicting one-day-ahead energy consumption. Compared to these studies in the literature, the SAMFOR model was very competitive and effective. The power of the hybrid approach comes from taking advantage of a linear model and a nonlinear model, in which an optimization algorithm was applied to fine-tune the configuration of the proposed model. Notably, in the proposed hybrid model, the FA was used to optimize the hyperparameters of the SVR. Moreover, appropriate inputs used for prediction were analyzed and selected via three sensitivity analyses. Thus, the SAMFOR model was an effective model for forecasting short-time energy consumption. The one-day-ahead energy consumption prediction results provide building owners, building managers, and network operators with insights and references to improve energy efficiency. Particularly, the operating schedule of appliances, lighting systems, and the air-conditioning system can be optimized and shifted to reduce energy costs.

## Conclusions

Sustainable development of energy is an important concern for many countries. Energy use reduction in buildings is beneficial to society in terms of economy and ecology. Challenges of energy consumption prediction include (1) selecting an appropriate value of lag for time-series energy use prediction in buildings, (2) identifying an appropriate input set for a prediction, (2) setting parameters of the prediction model, (3) selecting an appropriate size of the learning data for a prediction, and (4) optimizing the parameters of the prediction model.

This study proposed a hybrid artificial intelligence prediction model for forecasting time-series energy consumption in buildings toward sustainable development. The proposed model, namely SAMFOR, was constituted by the seasonal autoregressive integrated moving average (SARIMA), support vector regression (SVR), and firefly algorithm (FA). A large dataset of hourly energy consumption collected from buildings in Vietnam was used to train and test the performance of the proposed model. The proposed model achieved a great performance in predicting one-day-ahead energy consumption in the 30-min intervals in buildings. The accuracy measures by the SAMFOR model were 1.77 kWh in the root-mean-square-error, 5.02 kWh in the mean absolute error, 9.56% in the mean absolute percentage error, and 0.914 in the correlation coefficient in the test phase.

The hybrid SAMFOR model can take advantage of a linear model and a nonlinear model, in which an optimization algorithm was applied to fine-tune the configuration of the proposed model. Moreover, appropriate inputs used for prediction were analyzed and selected via three sensitivity analyses. Thus, the SAMFOR model was an effective model for forecasting short-time energy consumption. The proposed SAMFOR model improved the accuracy from 7.0 to 42.3 kWh in the RMSE as compared to baseline models of the SARIMA, SVR, and RF models. The results confirmed the effectiveness of the SAMFOR model in predicting energy consumption in buildings.

The first contribution of this work is the proposed effective prediction model in accurately forecasting the one-day-ahead energy consumption in buildings. The proposed hybrid model takes advantages of a linear model and a nonlinear model, in which an optimization algorithm was applied to optimize the proposed model. The second contribution is that the model can consider the temporal data (e.g., day of the week and hour of the day), weather data (e.g., outdoor temperature and humidity), and historical energy data as the inputs for the future energy use prediction in buildings. For practical contribution, the prediction results provide users with references to adjust their behavior and to improve energy efficiency.

As limitation, the model users need to have a background knowledge of artificial intelligence. Future work should develop a web-based decision support system, or an easy-to-use application based on the proposed prediction model to provide users with a convenience. The boundary conditions for developing this model are described as following: (1) the model was used for predicting one-day-ahead energy consumption in buildings, (2) the historical energy consumption, outdoor temperature, and temporal data were considered as inputs for the model, and (3) the model was developed and tested using the data in Vietnam. The proposed model can be expanded to apply for data in other countries.

## Data Availability

The datasets used and/or analysed during the current study available from the corresponding author on reasonable request.

## Abbreviations

SARIMA: Seasonal autoregressive integrated moving average
FA: Firefly algorithm
SVR: Support vector regression
SAMFOR: Seasonal Autoregressive integrated Moving average, and the Firefly-inspired Optimization algorithm and the support vector Regression
RF: Random forests
RMSE: Root-mean-square error
MAPE: Mean absolute percentage error
R: Correlation coefficient
ML: Machine learning
ARIMA: An autoregressive integrated moving average
AR: Autoregressive
AI: Artificial intelligence
ANNs: Artificial neural networks
LSSVR: Least-squares support vector regression
GA: Genetic algorithm
DE: Differential evolution
FDN: Feedforward deep networks
HVAC: Hourly heating ventilation air-conditioning
CART: Classification and regression tree
LR: Linear regression
LSSVM: Least-squares support vector machine
SCA: Sine cosine algorithm
RBF: Radial basis function
AIW: Adaptive inertia weight
FA-SVR: Firefly algorithm – support vector regression
LD: Learning data
OF: Objective function
*Y*: Historical energy consumption in 30-min intervals
*T*: Outdoor temperature
*H*: Outdoor humidity
*DoW*: Day of the week
*HoD*: Hour of the day
PSO: Particle swarm optimization
